# Peripheral Purinergic Modulation in Pediatric Orofacial Inflammatory Pain Affects Brainstem Nitroxidergic System: A Translational Research

**DOI:** 10.1155/2022/1326885

**Published:** 2022-03-11

**Authors:** Elisa Borsani, Andrea Ballini, Barbara Buffoli, Lorenzo Lo Muzio, Marina Di Domenico, Mariarosaria Boccellino, Salvatore Scacco, Riccardo Nocini, Vittorio Dibello, Rita Rezzani, Stefania Cantore, Luigi Fabrizio Rodella, Michele Di Cosola

**Affiliations:** ^1^Department of Clinical and Experimental Sciences, Division of Anatomy and Physiopathology, University of Brescia, Viale Europa 11, 25123 Brescia, Italy; ^2^Interdipartimental University Center of Research “Adaptation and Regeneration of Tissues and Organs (ARTO)”, University of Brescia, Viale Europa 11, 25123 Brescia, Italy; ^3^School of Medicine, University of Bari Aldo Moro, 70125 Bari, Italy; ^4^Department of Precision Medicine, University of Campania “Luigi Vanvitelli”, 80138 Naples, Italy; ^5^Faculty of Dentistry (Fakulteti i Mjekësisë Dentare-FMD), University of Medicine, Tirana, 1001 Tirana, Albania; ^6^Department of Clinical and Experimental Medicine, University of Foggia, 71122 Foggia, Italy; ^7^Department of Basic Medical Sciences, Neurosciences and Sensory Organs, University of Bari “Aldo Moro”, Bari 70124, Italy; ^8^Section of Ear Nose and Throat (ENT), Department of Surgical Sciences, Dentistry, Gynecology and Pediatric, University of Verona, Verona 37126, Italy; ^9^Department of Orofacial Pain and Dysfunction, Academic Centre for Dentistry Amsterdam (ACTA), University of Amsterdam and Vrije Universiteit Amsterdam, Amsterdam, Netherlands; ^10^Department of Interdisciplinary Medicine, Policlinico University Hospital of Bari, University of Bari Aldo Moro, 70124 Bari, Italy

## Abstract

Physiology of orofacial pain pathways embraces primary afferent neurons, pathologic changes in the trigeminal ganglion, brainstem nociceptive neurons, and higher brain function regulating orofacial nociception. The goal of this study was to investigate the nitroxidergic system alteration at brainstem level (spinal trigeminal nucleus), and the role of peripheral P2 purinergic receptors in an experimental mouse model of pediatric inflammatory orofacial pain, to increase knowledge and supply information concerning orofacial pain in children and adolescents, like pediatric dentists and pathologists, as well as oro-maxillo-facial surgeons, may be asked to participate in the treatment of these patients. The experimental animals were treated subcutaneously in the perioral region with pyridoxalphosphate-6-azophenyl-2′,4′-disulphonic acid (PPADS), a P2 receptor antagonist, 30 minutes before formalin injection. The pain-related behavior and the nitroxidergic system alterations in the spinal trigeminal nucleus using immunohistochemistry and western blotting analysis have been evaluated. The local administration of PPADS decreased the face-rubbing activity and the expression of both neuronal and inducible nitric oxide (NO) synthase isoforms in the spinal trigeminal nucleus. These results underline a relationship between orofacial inflammatory pain and nitroxidergic system in the spinal trigeminal nucleus and suggest a role of peripheral P2 receptors in trigeminal pain transmission influencing NO production at central level. In this way, orofacial pain physiology should be elucidated and applied to clinical practice in the future.

## 1. Introduction

Orofacial inflammatory pain is a universal healthcare complaint in pediatric patients and a major concern of national public health [1]. In general, orofacial pain results from two pathological processes: (1) tissue injury and inflammation (nociceptive pain) or (2) a primary lesion or dysfunction of the nervous system (neuropathic pain) [2, 3].

Extracellular adenosine triphosphate (ATP) has been detected at high concentrations in injured tissues during acute inflammation state, whether in experimental animals or in humans [4–6]. ATP can act as an extracellular signalling molecule [7, 8], influencing various biological functions, and stimulating nociceptors to initiate pain sensation by inducing the synthesis and release of proinflammatory cytokines and nitric oxide (NO) [9, 10]. Because of short half-life of NO and its highly reactive nature, most studies have focused on the analysis of its synthesizing enzyme, nitric oxide synthase (NOS). To date, three distinct NOS isoforms are known, named constitutive neuronal NOS (nNOS), endothelial NOS (eNOS), and inducible NOS (iNOS) [11, 12]. Some studies have suggested that NOS/NO may play a role in pathological pain states [13, 14]. In this regard, nNOS activity appears to influence pain transmission [10] while iNOS is expressed only in pathological conditions and is induced by proinflammatory cytokines and/or endotoxins [15]. A specific population of nNOS-positive neurons mainly mediate nociception [2]. Peripheral inflammation has been shown to alter the expression of both nNOS and iNOS in the spinal cord [16, 17], suggesting that the NO level in the spinal cord is closely regulated during inflammation. Moreover, some studies revealed a role of NO in the mesencephalic trigeminal nucleus for proprioception [18, 19]. Nevertheless, few studies have examined the role of the NO pathway at trigeminal level in inflammatory states [20, 21]. Lee and coworkers [22] observed a time-dependent increase in nNOS and iNOS protein expression in the spinal trigeminal nucleus following capsaicin injection in the masseter muscle and a significant attenuation of hypersensitivity after the pretreatment with NOS inhibitors in rats.

The spinal trigeminal nucleus, area receiving somatosensory inputs from the orofacial district, is subdivided into three parts, i.e., subnucleus caudalis (Sp5C), interpolaris (Sp5I), and oralis (Sp5O). The characterization of trigeminal pain pathways in inflammatory states is an important biological and clinical question for the development of new therapeutic strategies, but the role of the three subnuclei in the trigeminal nociceptive mechanisms is not yet well defined. Nevertheless, some studies showed that the most caudal part of the trigeminal sensory complex, i.e., Sp5C, is the essential projection site for the nociceptive orofacial inputs [23] but NOS proteins did not result in significant changes at longer time points [24].

ATP acts through P2 receptors that are subdivided into P2X and P2Y families. They are purinoreceptors classified into G-protein-coupled receptors P2Y and ATP-gated cation channels, so-called P2X receptors. In spinal and trigeminal ganglia, P2X [25, 26] and P2Y receptors are present [27].

In this study, pyridoxalphosphate-6-azophenyl-2′,4′-disulphonic acid (PPADS) was used, a wide range P2 receptor antagonist [28, 29]. The receptors more sensitive to PPADS are P2X_1_, P2X_2_, P2X_3_, and P2X_5_. Although all of them are present in the nervous system, P2X_1_, P2X_3_, and P2X_5_ receptors have been specifically described in trigeminal ganglia neurons [30, 31]. Besides, P2X_4_, P2X_7_, and P2Y_1_ receptors, less sensitive to PPADS, have been described in sensory ganglia and at presynaptic terminals [30].

Orofacial pain conditions represent a challenge to pediatric clinicians because of the disease complexity and unclear etiology. However, the defined mechanisms of pain management are still largely unknown.

With this premise, the aim of the present research was to investigate the relationship among inflammation, peripheral purinergic receptors, and central nitric oxide alteration in a mouse model of pediatric orofacial inflammatory pain, evaluating the alteration of the nitroxidergic system in the trigeminal spinal nucleus.

## 2. Materials and Methods

### 2.1. Animals

Experiments were carried out on a total number of 60 C57BL/6J male mice (20-25 gr. Harlan, Italy), and, in particular, 48 for immunohistochemistry and remaining 12 for western blotting (WB). The animal model used was around 6 weeks of age, which can be considered, according to literature, representative of pediatric patients [32].

To minimize the circadian variations, the animals were housed in individual cages with food and water *ad libitum* and kept in an animal house at a constant temperature of 22°C with a 12 h alternating light-dark cycle. The experiments were performed between 08:00 h and 12:00 h. All effort was made to minimize animal suffering, and the number of animals used agreed with the good animal practice (GAP). The experimental procedures were approved by the Italian Ministry of Health (n° 105/2011–B) and in accordance with the ethical standards of the Helsinki Declaration and the principles presented in the “Guidelines for the Use of Animals in Neuroscience Research” of the Society for Neuroscience.

The animals were subdivided into 4 experimental groups of 15 animals each: the first group was injected with saline (control, CTR); the second group was injected with PPADS (PPADS); the third group was injected with formalin (FORM); the fourth group was treated with PPADS and after 30 minutes injected with formalin (PPADS+FORM).

### 2.2. Injection Site and PPADS Treatment

The formalin and PPADS injections were performed subcutaneously into the right upper lip, just lateral to the nose through a 27-gauge needle into the right vibrissa pad as quickly as possible, with only minimal animal restraint.

PPADS tetrasodium salt (Sigma-Aldrich, Milan, Italy) was dissolved in saline and used at dose 25 mg/kg (0.01 ml/10 g), according to Gourine et al. [33], Martucci et al. [10], and our previous experience in this field [13].

### 2.3. Nociceptive Behavioral Response

The formalin test was made by injecting 2.5% formalin (FORM) according to Luccarini et al. [34]. Following formalin injection, all animals were immediately placed in the test box for a 60 minutes observation period. A nociceptive score was determined measuring the number of seconds that the animals spent rubbing the injected area with the ipsilateral forepaw or hindpaw. The recording time was divided into 20 blocks of 3 minutes. A video camera was used to record the grooming response.

### 2.4. Immunohistochemical Analysis

The nNOS and iNOS expressions were evaluated at 3, 6, 12, and 24 hours (h) after formalin injection.

All mice were anaesthetized with Zoletil (60 mg/kg i.p., Verbatic, France) and transcardially perfused with saline followed by 40 ml of 4% paraformaldehyde in phosphate buffer 0.1 M pH 7.4. After fixation, the brainstem of each animal was removed, postfixed in 4% paraformaldehyde in phosphate buffer for 2 h, and cryoprotected overnight in 30% sucrose at 4°C. Frozen serial transverse sections (40 *μ*m thick) of all the brainstem were placed in TBS (Tris-Buffer-Saline) solution. Alternate sections were processed immunohistochemically or toluidine blue-stained for morphological control.

Briefly, the first series of sections were incubated in normal goat serum (10% in phosphate-buffered saline containing 0.1% Triton X-100) for 60 minutes and then incubated in rabbit polyclonal primary antiserum directed against nNOS (1 : 500, Chemicon, USA) or iNOS (1 : 500, Chemicon, USA) diluted in phosphate-buffered saline containing 3% normal goat serum and 0.1% Triton X-100, for 24 h at 4°C. After incubation in the primary antiserum, the sections were sequentially incubated in biotinylated goat anti-rabbit immunoglobulins and avidin-biotin-peroxidase complex (Vector Labs., Burlingame, CA, USA). The reaction product was visualized using hydrogen peroxide and diaminobenzidine (Sigma, St. Louis, MO, USA) as chromogen. The immunohistochemical control was performed by omitting the primary antibody, in the presence of isotype-matched IgGs, and performing preadsorption assay using the related peptide and gave negative results.

The distribution of the labelled cells of all animals was charted with the aid of an image analyzer (Immagini & Computer, Milan, Italy).

### 2.5. Colocalization Immunofluorescence Assay

Double immunofluorescence aided the morphological identification of neurons through colocalization of nNOS or iNOS with NeuN (a nuclear marker of neurons).

Frozen floating sections, obtained using the procedure described above, of the brainstem were processed for double immunofluorescence. Briefly, the sections were incubated in bovine serum albumin (BSA, Sigma-Aldrich, Saint Louis, USA) blocking solution (5% BSA, 0.25% Triton X-100 in TBS 1%) and then were incubated in mouse monoclonal primary antiserum against NeuN (1 : 100, Chemicon, Temecula, CA, USA) with rabbit polyclonal primary antiserum against nNOS (1 : 500, Chemicon, USA) or iNOS (1 : 500, Chemicon, USA) diluted in TBS containing 3% BSA and 0.1% Triton X-100, for 24 h at +4°C. After incubation in the primary antibodies, the sections were sequentially incubated with appropriated fluorescent secondary antibodies diluted in TBS (1 : 200, Alexa-Fluor 488, green fluorescent dye and Alexa-Fluor 555, red fluorescent dye; Invitrogen, Carlsbad, CA, USA). The immunofluorescence control was performed by omitting the primary antibody and incubating the sections with nonimmune rabbit serum. All floating sections were placed on slides and finally mounted using a special mounting medium (UltraCruz™ Mounting Medium, Santa Cruz Biotechnology, Santa Cruz, CA) with DAPI (4′, 6-diamidino-2-phenylindole). The colocalization was evaluated on digital images acquired with laser scanning confocal microscopy (LSM 510, Zeiss, Germany).

### 2.6. Western Blotting Analysis

The nNOS and iNOS expressions were evaluated at 3 h after formalin injection. All mice were anaesthetized with Zoletil (60 mg/kg i.p.) and sacrificed by cervical dislocation. The brainstem in the region of the spinal trigeminal nucleus of each animal was removed and immediately frozen in liquid nitrogen and stored at -80°C until the NOS content expression assay. On the day of NOS determination, tissues were defrosted at room temperature, weighed, diluted in lysis buffer (Tris HCl pH 8 50 mM, NaCl 150 mM, Triton 1% 100 *μ*l/ml, PMSF 0.6 mM e aprotinina 1 *μ*g/ml), homogenized, and centrifuged at 13000g at 4°C for 2 minutes. After protein assay, the supernatant was diluted in Laemmli buffer (0.3 M Tris–HCl, pH 6.8, containing 10% SDS, 50% glycerol, 5% dithiothreitol, and 0.05% bromophenol blue) to obtain 40 *μ*g of proteins. The proteins were loaded onto an 8% SDS–polyacrylamide gel and then transferred onto a nitrocellulose membrane (Biosciences, Uppsala, Svezia) for 1 h at 4°C. The membrane was blocked with 5% BSA in TBST (20 mM Tris–base, pH 7.6, 137 mM NaCl, and 0.1% Tween 20) at 4°C overnight. The next day, it was incubated with primary polyclonal antibody directed against mouse nNOS (Cayman Chemical, Ann Arbor, MI, USA) diluted 1 : 200 or iNOS (Santa Cruz, Biotechnology, Santa Cruz, CA) diluted 1 : 500 in blocking solution (1% serum albumin bovine) for 2 h at room temperature. The nitrocellulose membrane was also probed with a polyclonal anti-*β*actin antibody (1 : 3000; Cytoskeleton Inc., Denver; CO, USA) as loading controls. After two washing in TBST buffer, the blot was incubated with biotinylated goat anti-rabbit immunoglobulins (Vector Labs., Burlingame, CA, USA) for 1 h at room temperature. Subsequently, the blot was detected with the addition of avidin-biotin-peroxidase complex (Vector Labs., Burlingame, CA, USA). The reaction product was visualized using hydrogen peroxide and diaminobenzidine (Sigma, St. Louis, MO, USA) as chromogen.

### 2.7. Data Analysis

Behavioral analysis was made for 1 h after the formalin injection by three investigators (E.B., A.B, and S.C.) who were blinded to the group of animal assignment. The animal data were analyzed and compared by repeated ANOVA (analysis of variance) measurements followed by Tukey's post-test. The density of nNOS- and iNOS-positive neurons in the brainstem was evaluated by immunohistochemical analyses in Sp5C, Sp5I, and Sp5O using a quantitative method by blinded researchers. The neurons were recognized by their morphological characteristics [10] and supported by a colocalization immunofluorescence assay with a NeuN. nNOS- and iNOS-positive cell counts were made in all the processed sections at a final 200x magnification. Total counts were taken from each section and assigned to specific components of the brainstem trigeminal complex. Cytoarchitecturally identified regions of the spinal trigeminal nucleus including Sp5C, Sp5I, and Sp5O were examined for nNOS- and iNOS-positive cells. Rostrocaudal levels of these subnuclei were referred to as bregma according to coordinates provided by Franklin and Paxinos [35]. Moreover, a set of serial transverse 40 *μ*m sections stained with toluidine blue (Sigma, St. Louis, MO, USA) was prepared to identify the area of Sp5 subnuclei better. We analyzed the following according to the bregma coordinates: Sp5O sections were collected from -5.68 mm to -6.48 mm, Sp5I from -6.48 mm to -7.48 mm, and Sp5C from -7.48 mm to -8.48 mm. Immunoreactive bands of western blot analysis were analyzed using a computer-based densitometry image program. Grey levels were evaluated as integrated optical density (IOD) with an image analysis program (Image-Pro Premier 9.1, Milan, Italy).

The immunohistochemical and immunoblotting data were analyzed and compared by repeated-measures ANOVA followed by Tukey's post-test.

## 3. Results

### 3.1. Behavior Analysis

**Figure 1:**
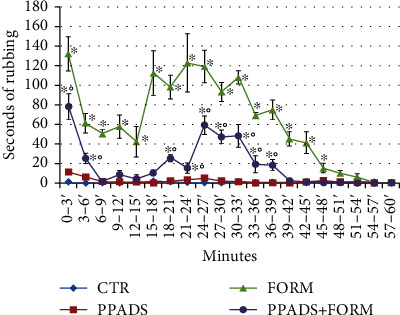
Time course of face-rubbing activity observed after subcutaneous injection of saline (CTR), formalin (FORM), PPADS (25 mg/kg), and formalin (PPADS+FORM) into the right upper lip. The mean number of seconds that each mouse spent rubbing was plotted for each 3 minutes block over the 60 minutes postinjection observation period. The experiments were performed in triplicate. Data represent mean ± S.D.; ^∗^*p* < 0.05 versus CTR animals; °*p* < 0.05 versus FORM animals.

The evaluation of nociceptive threshold was performed for 1 h after the injection. Control animals showed only face-grooming episodes for full analysis time (Figure 1).

PPADS-treated animals displayed a nociceptive score not significantly different from control animals (Figure 1).

Formalin-injected animals showed sustained face-rubbing episodes with vigorous face-wash strokes directed to the perinasal area with the ipsilateral and, sometimes, contralateral forepaw. The forepaw was often accompanied in its movements by the hindpaw. This nociceptive response presented a typical biphasic time course interspersed with a period of quiescence (10-15 minutes): (1) an early and short-lasting first period of activity (3–5 minutes) and (2) a second prolonged (20–45 minutes) phase (Figure 1). Animals pretreated with PPADS and injected with formalin showed a significant decrease of nociceptive score with respect to the formalin animals. Particularly, they presented a less pronounced early phase and a less marked and lasting second rubbing period (Figure 1).

### 3.2. Immunohistochemical Evaluation

**Figure 2:**
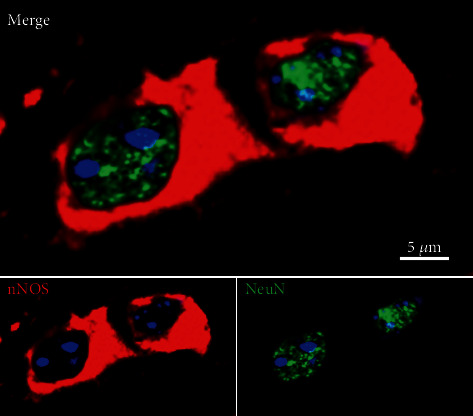
Double-label confocal images of spinal trigeminal nucleus stained neurons for NeuN (green) and nNOS (red). The nuclei were stained in blue (DAPI). Bar: 5 *μ*m.

Double immunofluorescence aided the morphological identification of neurons through colocalization of nNOS or iNOS with NeuN (Figure 2).

**Figure 3:**
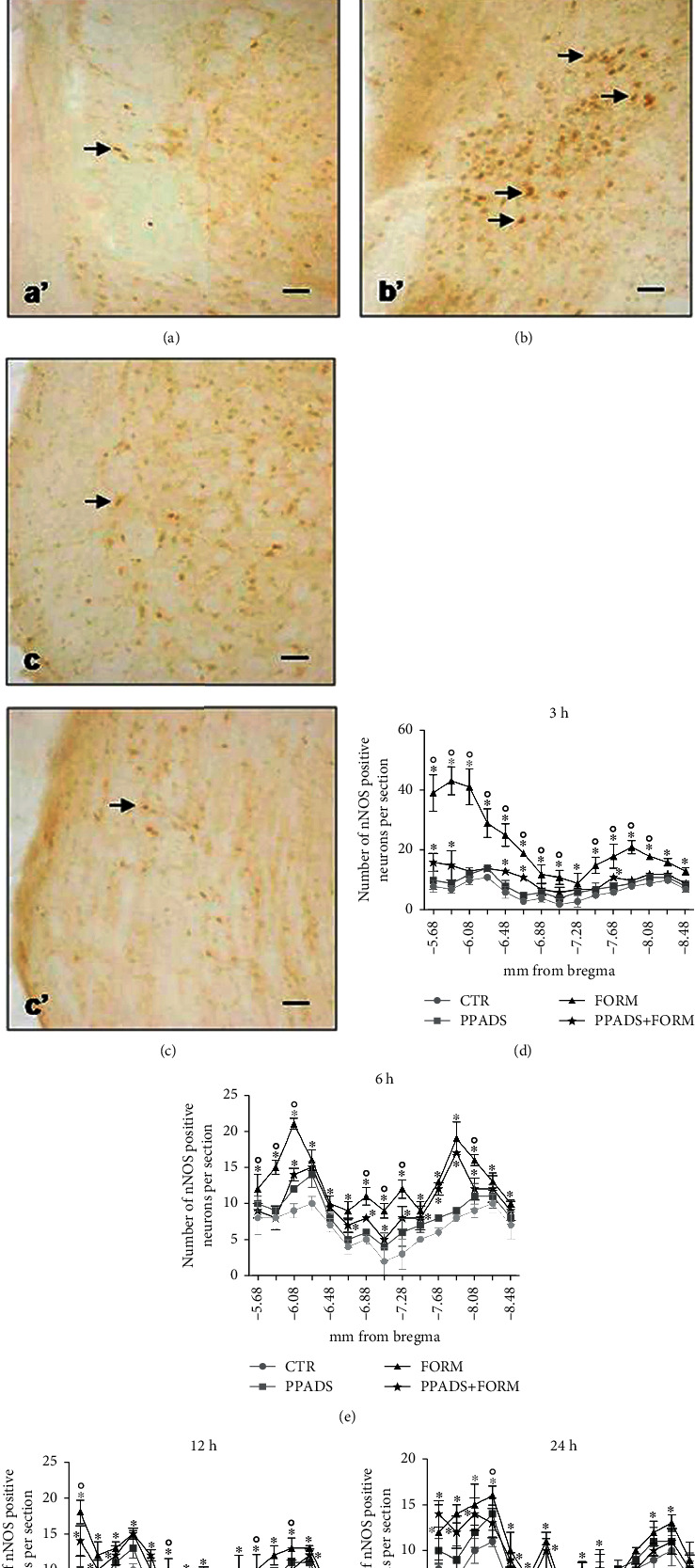
nNOS-positive neurons in the ipsilateral spinal trigeminal nucleus: (a) subnucleus caudalis (Sp5C) of saline-treated animals (CTR), (b) Sp5C of formalin-treated animals (FORM), (c) Sp5C of PPADS and formalin-treated animals (PPADS+FORM), (a′) subnucleus oralis (Sp5O) of saline-treated animals (CTR), (b′) Sp5C of formalin-treated animals (FORM), and (c′) Sp5C of PPADS and formalin-treated animals (PPADS+FORM). Arrows indicate nNOS-positive neurons. Bar 50 *μ*m. Time course of nNOS immunopositive neurons in the ipsilateral spinal trigeminal nucleus in saline-treated animals (CTR), formalin-treated animals (FORM) and PPADS (25 mg/kg), and formalin-treated animals (PPADS+FORM) after (d) 3, (e) 6, (f) 12, and (g) 24 h from formalin injection. The experiments were performed in triplicate. Data represent mean ± S.D.^∗^*p* < 0.05 vs. CTR; °*p* < 0.05: FORM vs. PPADS+FORM.

The time course of nNOS displayed a rapid increase of protein staining at 3 h in FORM animals, in Sp5C, Sp5I, and Sp5O (Figures 3(b), 3(b′), and 3(d)). Over 24 h, nNOS gradually decreased towards control values both for Sp5C, Sp5I, and Sp5O (Figures 3(e)–3(g)). The PPADS treatment partially limited the increase of nNOS in the trigeminal nucleus reaching a statistically decrease (Figures 3(c), 3(c′), and 3(d)–3(g)).

**Figure 4:**
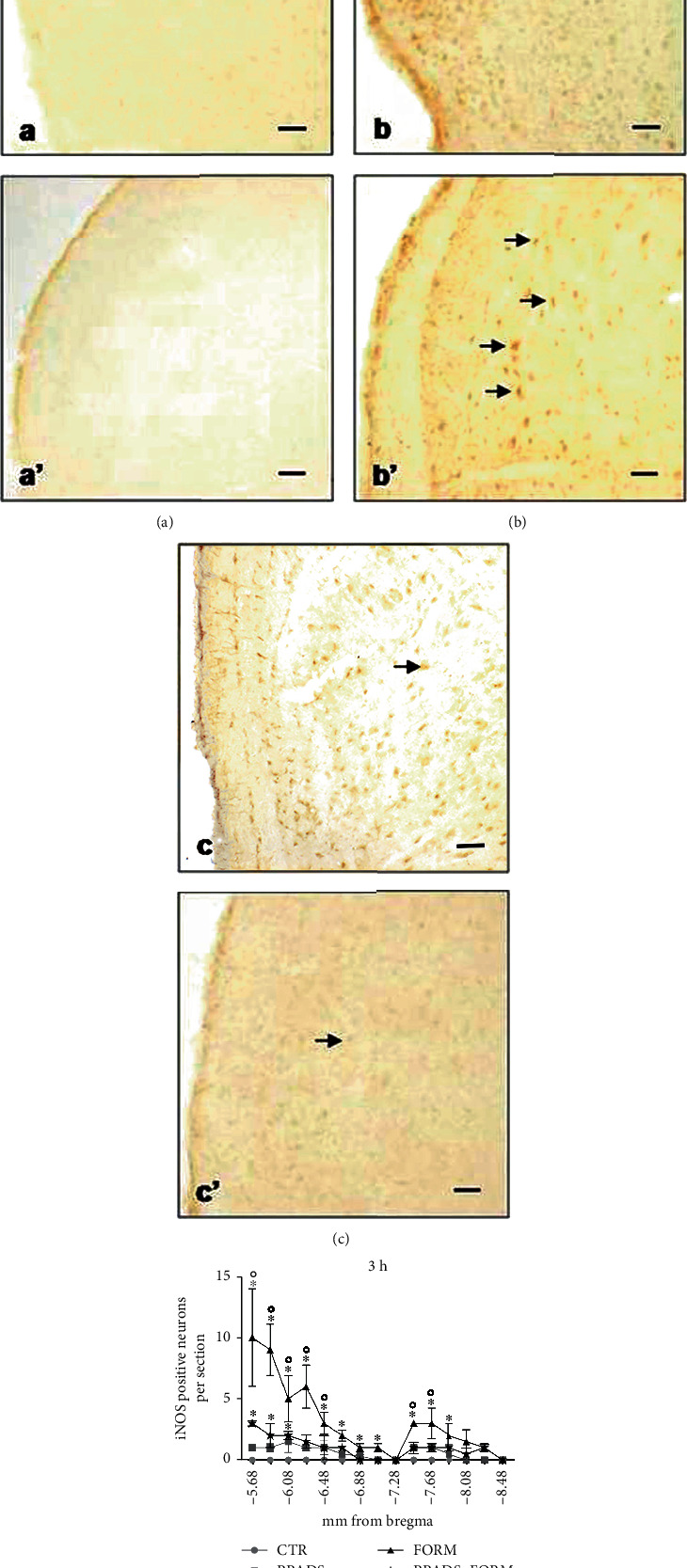
iNOS-positive neurons in the ipsilateral spinal trigeminal nucleus: (a) subnucleus caudalis (Sp5C) of saline-treated animals (CTR), (b) Sp5C of formalin-treated animals (FORM), (c) Sp5C of PPADS and formalin-treated animals (PPADS+FORM), (a′) subnucleus oralis (Sp5O) of saline-treated animals (CTR), (b′) Sp5C of formalin-treated animals (FORM), and (c′) Sp5C of PPADS and formalin-treated animals (PPADS+FORM). Arrows indicate iNOS-positive neurons. Bar 50 *μ*m. Statistical evaluation of immunopositive neurons in the ipsilateral trigeminal nucleus in saline-treated animals (CTR), formalin-treated animals (FORM) and PPADS (25 mg/kg), and formalin-treated animals (PPADS+FORM) after 3 h from formalin injection. The experiments were performed in triplicate. Data represent mean ± S.D.^∗^*p* < 0.05 vs. CTR; °*p* < 0.05: FORM vs. PPADS+FORM.

The time course of iNOS showed a rapid increase of protein staining at 3 h in FORM animals in Sp5C (Figure 4(d)). Over 24 h, iNOS immunostaining was low reaching control values (data not shown). The PPADS treatment partially limited the increase of iNOS reaching a statistically decrease (Figures 4(c), 4(c′), and 4(d)).

At 3 h, the expression of nNOS and iNOS was maximum in our experiment. At this time point, the nNOS and iNOS immunoreactivities were localized in the cytoplasm of neurons in all areas of the trigeminal subnuclei appearing as brown staining while the nuclei were unstained. In control and PPADS-treated animals, the number of nNOS-positive neurons in Sp5C, Sp5I, and Sp5O was very low without any significant difference between the sections (Figures 3(a), 3(a′), and 3(d)) while iNOS staining was not found (Figures 4(a), 4(a′), and 4(d)). On the contrary, in formalin-injected animals, the number of nNOS-positive cells greatly increased in the Sp5O and in the Sp5C areas compared to control and PPADS-treated animals (Figures 3(b), 3(b′), and 3(d)). The formalin-related increase of positivity was found mainly in Sp5O and in the ipsilateral side. In Sp5O, iNOS-positive neurons increased too (Figures 4(b′) and 4(d)). On the other hand, in animals pretreated with PPADS and injected with formalin, significantly fewer nNOS-positive neurons were observed bilaterally in all three parts of the spinal trigeminal nucleus compared to the formalin group (Figures 3(c), 3(c′), and 3(d)). Moreover, a recovery to normal iNOS staining was also reached (Figures 4(c), 4(c′), and 4(d)).

### 3.3. Western Blotting Analysis after PPADS Treatment at 3 h: nNOS

The nNOS expression in the brainstem decreased after PPADS treatment.

**Figure 5:**
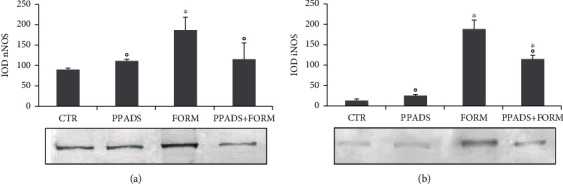
Statistical evaluation of nNOS (a) and iNOS (b) expression in the brainstem in saline-treated animals (CTR), PPADS-treated animals (PPADS), formalin-treated animals (FORM), and PPADS- and formalin-treated animals (PPADS+FORM) after 3 h from formalin injection. The experiments were performed in triplicate. Values are mean ± S.D. and represent the IOD (integrated optical density); ^∗^*p* < 0.05 vs. CTR animals; °*p* < 0.05 vs. FORM animals.

In control- and PPADS-treated animals, the nNOS expression was present and moderate (Figure 5(a)).

On the contrary, in formalin-injected animals, nNOS was overexpressed in the brainstem neurons compared to control animals (Figure 5(a)).

The pretreatment with PPADS showed a decrease of a nNOS expression in neurons (Figure 5(a)).

### 3.4. Western Blotting Analysis after PPADS Treatment at 3 h: iNOS

The iNOS expression in the brainstem decreased after PPADS treatment. In control and PPADS-treated animals, the iNOS expression was not found (Figure 5(b)).

On the contrary, in formalin-injected animals, iNOS expression in neurons was increased compared to control animals (Figure 5(b)).

## 4. Discussion

The results of this study suggest the correlation between the nitroxidergic system in the brainstem and peripheral P2 receptor modulation in orofacial inflammatory pain transmission contributing to the insight of this pathology [36].

Our results showed that the local application of PPADS in the inflamed site produces a reduction in pain-related behavior, as reported also in Borsani et al. [13]. P2 receptors are activated by ATP released in inflamed tissue promoting pain sensation. In this regard, acute peripheral inflammation induces an increase of extracellular ATP at the sites of tissue injury [4, 5] with consequent excessive activation of P2X receptors on primary sensory axons. An elevated P2X receptor activity can also result from the enhanced expression of this receptor in inflamed tissue and can contribute to abnormal pain responses associated with inflammatory injuries [37]. Indeed, in our previous experience, animals pretreated with A-317491, a P2X3, and P2X2/3 receptor antagonist and then injected with formalin in the perioral area had a statistically less pronounced early phase and a delayed second rubbing period compared to animals treated only with formalin [13]. These receptors are also involved in chronic inflammatory conditions such as rheumatoid arthritis [38]. Moreover, P2Y_14_ receptor in trigeminal ganglia may contribute to the maintenance of orofacial inflammatory pain [39].

Furthermore, we showed a modulation of the nitroxidergic system in the spinal trigeminal nucleus, at Sp5C and Sp5O level. We observed an increase in nNOS immunostaining in the superficial laminae especially at 3 h decreasing at 24 h and an increase in iNOS immunostaining at 3 h after formalin injection. These observations suggest a role for NO in nociception also in the orofacial system at central level. In literature, there are poor knowledge about the relationship between orofacial nociception and NO [35, 40]. Nevertheless, it has been demonstrated that nNOS [17] and iNOS [41, 42] might play a critical role in central mechanisms of the development and/or maintenance of inflammatory pain, supporting our results. Other studies demonstrated the nNOS expression at trigeminal level not only in mammals [43–46] but also in birds and in reptiles [47]. The results of Fan and coworkers [48] displayed that NO plays an active role in both peripheral and central processing of nociceptive information following chronic tooth inflammation.

The significant decrease of nNOS and iNOS that we observed after the pretreatment with PPADS suggests an important role of NO-ATP in orofacial nociceptive transmission. Also interestingly, acute peripheral inflammation induces an increase of extracellular ATP at the sites of tissue injury [4, 5], with consequent excessive activation of P2X receptors on primary sensory axons. In addition, some works regarding peripheral inflammation have revealed that NOS expression in the central nervous system is differentially regulated, because of the target organ and the proinflammatory agent employed [49, 50], suggesting also a possible antinociceptive role.

Other studies have shown that the most caudal part of the trigeminal sensory complex, i.e., Sp5C, is the essential projection site for the nociceptive orofacial inputs [23, 24, 51]. In this specific subnucleus, our data showed an increase in the number of nNOS- and iNOS-positive neurons after formalin injection in the ipsilateral part and its decrease with PPADS pretreatment, demonstrating a correlation between peripheral purinergic receptor modulation and NO production in pain perception. We also observed an increase of nNOS- and iNOS-positive neurons in Sp5O too. These results suggest a role in nociceptive transmission of perioral area for both the subnuclei, and they are corroborated by other experimental observations reported in literature. Electrophysiological studies performed in rat and monkey [52–54] indicate that one or several of the three rostral divisions of the trigeminal sensory complex, i.e., the nucleus principalis (Pr5), Sp5O, and Sp5I, may also be involved in the transmission of orofacial pain. Some works focused the attention on the role of Sp5O as involved in intraoral nociceptive stimulation [55], even if it has been reported also a possible involvement of Sp5C in this area [56]. On the other hand, other experiments suggest the role of Sp5O in perioral nociceptive mechanisms [29]. In fact, abundant data indicate that the rostral relay for some oral/perioral nociceptive molecules is in Sp5O. The Sp5O lesions observed in humans or performed in animals induced a significant decrease in the nociceptive sensations or behaviors triggered by intraoral [56] but also perioral noxious stimuli [57].

Altogether, these data suggest a specific role and specialization of Sp5O and Sp5C in the processing of the nociception, confirming our previous results, even if some authors indicated only the Sp5C involved in formalin perioral stimulation [58, 59]. In particular, Sp5O is activated in transient nociception, while Sp5C in sustained nociception [60, 61].

## 5. Conclusions

In conclusion, our results suggest a key role for the endogenous ATP which can contribute at peripheral level to induct acute inflammatory pain processing. Moreover, we have demonstrated that the events after inflammatory induction involve ATP and NO, influencing the nociceptive pathways in the central nervous system. Based on our results, the PPADS could represent a therapeutic tool for the orofacial inflammatory pain in pediatric population. Large multicenter trials are required in order to study the biological behavior and formulate treatment strategies in the management of the same.

## Data Availability

The data used to support the findings of this study are included within the article.
